# C4, the Pathogenic Determinant of *Tomato Leaf Curl Guangdong Virus*, May Suppress Post-transcriptional Gene Silencing by Interacting With BAM1 Protein

**DOI:** 10.3389/fmicb.2020.00851

**Published:** 2020-05-05

**Authors:** Zhenggang Li, Zhenguo Du, Yafei Tang, Xiaoman She, Xiaomei Wang, Yanhua Zhu, Lin Yu, Guobing Lan, Zifu He

**Affiliations:** ^1^Plant Protection Research Institute, Guangdong Academy of Agricultural Sciences, Guangzhou, China; ^2^Guangdong Provincial Key Laboratory of High Technology for Plant Protection, Guangdong Academy of Agricultural Sciences, Guangzhou, China

**Keywords:** *Tomato yellow leaf curl Guangdong virus*, C4, pathogenic determinant, PTGS, TGS, BAM1

## Abstract

*Tomato leaf curl Guangdong virus* (ToLCGdV) is a begomovirus associated with a Tomato yellow leaf curl disease (TYLCD) epidemic in Guangdong province, China. Being the least conserved protein among geminivirus proteins, the function of C4 during ToLCGdV infection has not been elucidated. In this study, the infectious clones of ToLCGdV and a ToLCGdV mutant (ToLCGdV_mC4_) with disrupted C4 ORF were constructed. Although ToLCGdV and ToLCGdV_mC4_ could infect *Nicotiana benthamiana* and tomato plants, ToLCGdV_mC4_ elicited much milder symptoms compared with ToLCGdV. To further verify the role of C4 in viral pathogenesis, C4 was expressed in *N. benthamiana* from *Potato virus X* (PVX) vector. The results showed that ToLCGdV C4 enhanced the pathogenicity of PVX and induced more severe developmental abnormalities in plants compared with PVX alone or PVX-mC4. In addition, ToLCGdV C4 suppresses systemic gene silencing in the transgenic *N. benthamiana* line 16c, but not local gene silencing induced by sense GFP in wild-type *N. benthamiana* plants. Moreover, C4 suppresses transcriptional gene silencing (TGS) by reducing the DNA methylation level of 35S promoter in 16c-TGS *N. benthamiana* plants. Furthermore, C4 could also interact with the receptor-like kinase (RLK) BARELY ANY MERISTEM 1 (BAM1), suggesting that C4 may suppress gene silencing by interfering with the function of BAM1 in the cell-to-cell spread of RNAi. All these results suggest that C4 is a pathogenic determinant of ToLCGdV, and C4 may suppress post-transcriptional gene silencing (PTGS) by interacting with BAM1.

## Introduction

*Geminiviridae* constitutes a large family of plant viruses with small, single-stranded and circular DNA (sscDNA) genomes encompassed within characteristic twinned icosahedral particles (Hanley-Bowdoin et al., 1999; Fauquet et al., 2008). Geminiviruses, which are transmitted by insects and could infect both dicots and monocots (John et al., 2001), have emerged as one of the most important groups of plant pathogens worldwide, especially in tropical and subtropical regions (Boulton, 2003; Varma and Malathi, 2003; Rojas et al., 2005; Mansoor et al., 2006; Fauquet et al., 2008; Navas-Castillo et al., 2011; Sattar et al., 2013). *Geminiviridae* includes nine genera *Becurtovirus*, *Begomovirus*, *Capulavirus*, *Curtovirus*, *Eragrovirus*, *Grablovirus*, *Mastrevirus*, *Topocuvirus*, and *Turncurtovirus* with different genome organization, host range, and insect vectors (Zerbini et al., 2017). Except the genus *Begomovirus*, geminivirus consists of monopartite component with 2.5∼3.0 kb in length (Zerbini et al., 2017).

Begomoviruses, transmitted by the whitefly vector *Bemisia tabaci*, are either monopartite or bipartite and infect only dicots. The DNA A virion-sense strand of bipartite begomovirus encodes the coat protein CP (AV1) and movement protein (AV2), which may both be involved in virus movement. The complementary-sense strand of DNA A component encodes the replication-associated protein (Rep, ORF AC1), transcriptional activator protein (TrAP, ORF AC2), replication enhancer protein (REn, ORF AC3) and C4 protein (ORF AC4). The DNA B component of bipartite begomovirus encodes two proteins, the nuclear shuttle protein (NSP, ORF BV1) on the virion-sense strand and the movement protein (MP, ORF BC1) on the complementary-sense strand (Hanley-Bowdoin et al., 1999). For both DNA A and DNA B, the bidirectionally arranged ORFs are separated by a long intergenic region (IR) which carries key elements for initiating viral replication and transcription (Hanley-Bowdoin et al., 1999). The genome of monopartite begomovirus encodes all information required for the viral infection and consists of a single ssDNA molecule which resembles the DNA A of bipartite begomovirus. However, an increasing number of monopartite begomoviruses have been found to require betasatellites for successful infection and symptom induction in some host plants (Zhou, 2013; Yang et al., 2019).

As one of the least conserved proteins among geminivirus proteins, the function of AC4/C4 protein varies among different geminiviruses. C4 is a major pathogenic determinant in curtoviruses and some monopartite begomoviruses, disruption of C4 reduces viral infections and symptom development (Stanley and Latham, 1992; Teng et al., 2010; Li et al., 2018). Transient expression of *Beet curly top virus* (BCTV) C4 or *Tomato leaf curl Yunnan virus* (TLCYnV) C4 contributes to PVX infection, and induces severe developmental abnormalities similar with phenotypes induced by virus infection in both *N. benthamiana* and *Arabidopsis* (Latham et al., 1997; Piroux et al., 2007; Mills-Lujan and Deom, 2010; Mills-Lujan et al., 2015; Mei et al., 2018b). However, AC4 of the bipartite begomovirus *Tomato golden mosaic virus* (TGMV) has no obvious effect on symptom development (Piroux et al., 2007). More and more studies have found that AC4/C4 induces plant abnormal development by regulating brassinosteroid (BR) signaling pathway through interaction with members of *Arabidopsis* SHAGGY-like protein kinase (AtSK) family (Piroux et al., 2007; Mills-Lujan and Deom, 2010; Deom and Mills-Lujan, 2015; Mills-Lujan et al., 2015; Bi et al., 2017; Mei et al., 2018a, b).

AC4/C4 also functions as a viral suppressor of RNA silencing (VSR) to suppress both post-transcriptional gene silencing (PTGS) and transcriptional gene silencing (TGS) (Vanitharani et al., 2004; Gopal et al., 2007; Yang et al., 2011; Fondong, 2013; Xie et al., 2013). AC4 encoded by *African cassava mosaic virus* (ACMV) and *Sri Lankan cassava mosaic virus* (SLCMV), but not *East African cassava mosaic Cameroon virus* (EACMCV) nor *Indian cassava mosaic virus* (ICMV), have been shown to suppress RNA silencing by specifically binding to the single-stranded miRNAs and siRNAs to block the miRNA-mediated cleavage of target mRNAs (Chellappan et al., 2004, 2005; Vanitharani et al., 2004; Fondong et al., 2007). A recent study also shows that *Tomato yellow leaf curl virus* (TYLCV) C4 interacts with the receptor-like kinases (RLKs) BARELY ANY MERISTEM 1 (BAM1) and its homolog BAM2, interfering with the function of BAM1 and BAM2 in the intercellular spread of RNAi (Rosas-Diaz et al., 2018). Moreover, *Mungbean yellow mosaic virus* (MYMV) AC4 also interacts with BAM1 at plasma membrane (PM), and the PM location of MYMV AC4 depends on S-palmitoylation (Carluccio et al., 2018). AC4/C4 can also reverse methylation-mediated TGS. *Cotton leaf curl Multan virus* (CLCuMuV) C4 have been shown to repress both PTGS and TGS by interacting with S-adenosyl methionine synthetase (SAMS) and inhibiting SAMS activity (Ismayil et al., 2018).

A group of begomoviruses, most of which are monopartite geminiviruses, infect tomato plants and are considered to be responsible for a devastating disease called tomato yellow leaf curl disease (TYLCD) which is a major limiting factor for tomato cultivation worldwide (Navas-Castillo et al., 2011). A new begomovirus which might be responsible for the epidemic of TYLCD in Guangdong province of China during the year 2004–2006 was isolated and named *Tomato leaf curl Guangdong virus* (ToLCGdV) (He et al., 2005). ToLCGdV is a monopartite begomovirus with 2744nt in length and encodes 6 proteins as the other monopartite geminiviruses. As expected, ToLCGdV CP encoded by virion-sense strand shares the highest amino acid identity compared with other begomoviruses, while C4 protein shares the least amino acid identity (He et al., 2005). As the huge diversity of AC4/C4, the function of ToLCGdV C4 is still unknown.

In this study, the infectious clones of ToLCGdV and ToLCGdV_mC4_ were constructed. ToLCGdV C4 is a pathogenic determinant by contributing to ToLCGdV and PVX infection. ToLCGdV C4 is also a VSR by suppressing systemic gene silencing but not local gene silencing. Moreover, ToLCGdV C4 could reverse methylation-mediated TGS. In addition, ToLCGdV C4 suppresses PTGS maybe by interacting with BAM1. These results demonstrate that ToLCGdV C4 plays an important role in virus infection by functioning as both pathogenic determinant and VSR.

## Materials and Methods

### Plasmid Construction

To construct ToLCGdV infectious clones pGreenII-1.3A-ToLCGdV which contains a 1.3-mer tandem repeat of ToLCGdV, the full-length sequence and a 0.8-kb fragment of ToLCGdV were amplified with primer pairs ToLCGDV-F1/R1 and ToLCGDV-F2/R2 (Supplementary Table 1), respectively, and introduced into *Sma*I-digested pGreenII (Hellens et al., 2000) by Seamless Cloning and Assembly (Takara). pGreenII-1.3A-ToLCGdV_mC4_ was generated by QuickChange^®^ site-directed mutagenesis (Agilent Technologies) with pGreenII-1.3A-ToLCGdV as templates.

C4 and the mutated C4 nucleotide sequences were amplified from pGreenII-1.3A-ToLCGdV or pGreenII-1.3A-ToLCGdV_mC4_ and introduced into pGR107 (Lu et al., 2003) to obtain PVX-C4 and PVX-mC4. pGD-C4-Myc was generated by inserting C4 sequence into pGD-Myc (Goodin et al., 2002). Subsequently, the sequence of C4-Myc was amplified and introduced into pGR107 to get PVX-C4-Myc.

For bimolecular fluorescence complementation (BiFC) assays, C4 was amplified and cloned into pSPYNE-35S and pSPYCE-35S split YFP destination vectors (Walter et al., 2004). For subcellular localization experiments, C4 was amplified, followed by cloning into pGDGm (Goodin et al., 2002; Fan et al., 2014) with standard protocols.

Primers used for plasmid construction in this study are listed in Supplementary Table 1.

### Plant Growth Conditions

*Nicotiana benthamiana* and tomato plants used in this study were grown in a climate chamber with a 13h/11h light/dark photoperiod at 24°C. *N. benthamiana* were agroinfiltrated at 5–6 leaf stage, and tomato plants were inoculated at 6–7 leaf stage.

### Western Blot Detection

For western blot detection, samples were taken and weighed, followed by ground in liquid nitrogen. Two volumes of 1 × SDS loading buffer was added and boiled for 10 min. After centrifugation for 10 min at 12 000 rpm, 20 μl supernatant was loaded per lane on the SDS-PAGE gel. Then proteins were transferred to the nitrocellulose membranes. The secondary antibody was purchased from Sigma-Aldrich, Inc., Alkaline phosphatase was visualized with NBT/BCIP (Sangon Biotech, Shanghai, China).

### Phylogenetic Analyses

AC4/C4 protein sequences were obtained by searching National Center for Biotechnology Information (NCBI) GenBank using the protein sequence of ToLCGdV C4. Phylogenetic trees were constructed with MEGA7 (Kumar et al., 2016) software using neighbor-joining method based on AC4/C4 protein sequences. 1000 bootstrap replicates were performed to obtain support for the identified phylogenetic relationships.

### Quantitative RT-PCR

Total RNA was extracted with Trizol reagent (Takara) and treated with RNase-free rDNase I (Takara) before reverse transcription reaction. Equal amount of treated RNA was subjected to reverse transcription using PrimeScript RT Reagent Kit (Takara). Real-time RT-PCR was performed with SYBR Premix Ex Taq II (Takara) using CFX96 Real-Time System (Bio-Rad, United States).

### GFP Imaging

To identify the suppressor activity of C4, 16c transgenic *N. benthamiana* plants (provided by Dawei Li lab) and 16c-TGS plants were used. 16c-TGS plants were generated as described (Buchmann et al., 2009). *Agrobacterium* infiltration was performed as described previously (Hamilton et al., 2002). *Tomato bushy stunt virus* (TBSV) p19 and an empty vector (pGD) were used as positive and negative controls, respectively. GFP fluorescence was observed under a long-wave UV lamp (Black Ray model B 100A; UV Products) and photographed using a Nikon D70 digital camera with a Y48 yellow filter.

### Confocal Microscopy

*Agrobacterium* strains harboring the corresponding plasmids were co-infiltrated into 5–6 leaf stage *N. benthamiana* plants. Samples were taken at 3 dpi and visualized under Zeiss LSM710 confocal microscope (Carl Zeiss 710, Germany). Excitation wavelengths were as follows: EYFP, 514 nm; RFP, 561 nm.

### Genomic DNA Isolation and Next Generation Sequencing-Based Bisulfite Sequencing PCR (BSP)

Genomic DNA was extracted with a cetyltrimethylammonium bromide (CTAB) method (Doyle and Doyle, 1987). Cytosine methylation level of the 35S promoter region was assayed by next generation sequencing-based BSP method as described previously (Gao et al., 2014, 2015; Pan et al., 2018). Primer pair used to amplify 35S promoter was described previously (Ismayil et al., 2018). PCR products of 35S promoter from different samples were quantified by Qubit 3.0 (Thermo Fisher), followed by bisulfite treatment with EZ DNA Methylation Gold Kit (Zymo Research). Then the products were subjected to adapter ligation by PCR amplification to generate barcoded libraries. Barcoded libraries from different samples were pooled together equally for standard pair-end sequencing with Illumina HiSeq PE150. The acquired data were processed with standard protocols (Li et al., 2010).

### Co-immunoprecipitation (Co-IP) Assays

Co-immunoprecipitation assays were performed according to the previously published protocols (Rubio et al., 2005; Hu et al., 2015). Inoculated *N. benthamiana* leaves were collected at 3 dpi to perform Co-IP assays with anti-c-Myc agarose affinity gel (Sigma Aldrich, United States).

## Results

### Phylogenetic Analysis of ToLCGdV C4

**FIGURE 1:**
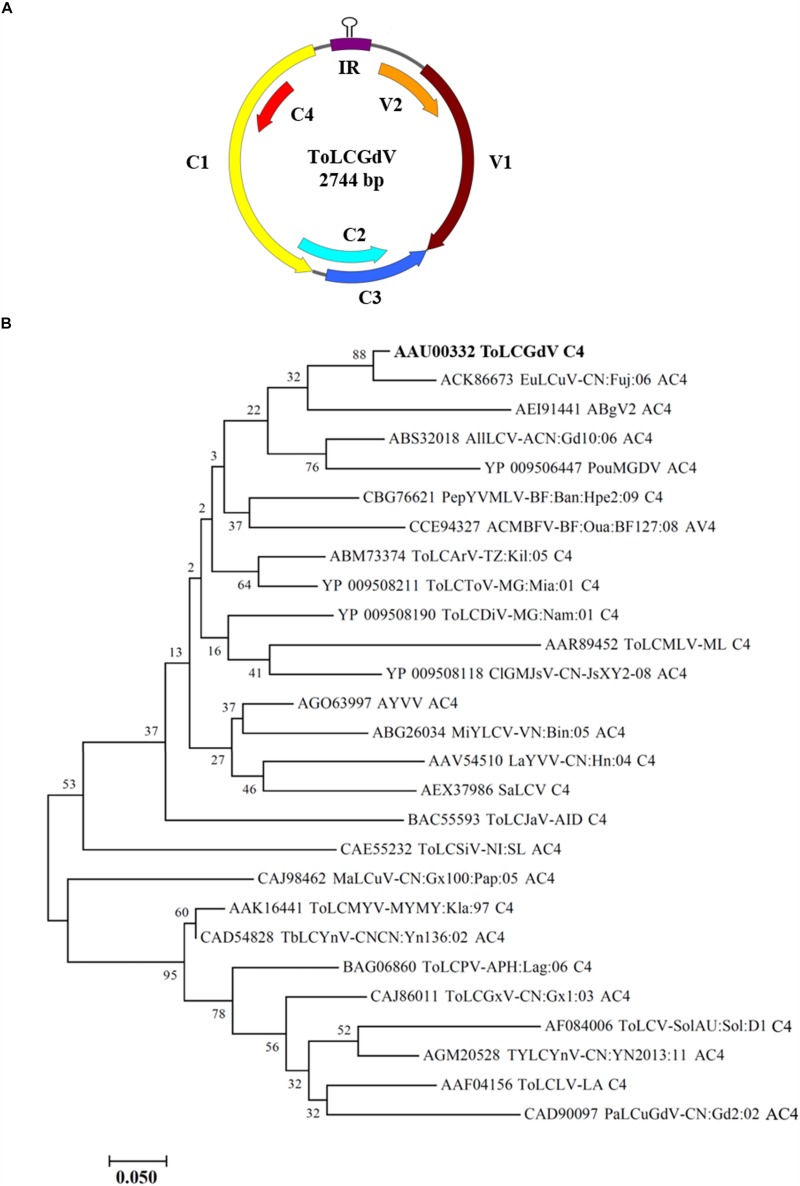
Schematic diagram of ToLCGdV genome and phylogenetic tree constructed from AC4/C4 protein sequences by neighbor-joining method. **(A)** Schematic diagram of ToLCGdV genome. Viral genes are indicated by filled arrows. IR, intergenic region. **(B)** Phylogenetic tree constructed from AC4/C4 protein sequences by neighbor-joining method. Twenty-seven AC4/C4 protein sequences were acquired from the GenBank. Full names and the origins of the begomoviruses used in the phylogenetic analyses are listed in Table 1. Numbers on the branches are bootstrap obtained from 1000 replicates. ToLCGdV C4 are marked in bold.

*Tomato leaf curl Guangdong virus* was firstly reported and named by He et al. (2005), and no satellite molecules were found to be associated with ToLCGdV. ToLCGdV contains 2744 nucleotides and encodes 6 proteins, V1, V2, C1, C2, C3, and C4 (Figure 1A). As the least conserved protein among geminiviruses, the variety of C4 is one of the main reasons that responsible for the diversity of geminiviruses. To explore the relationship of ToLCGdV C4 with other begomoviruses AC4/C4, multiple AC4/C4 protein sequences of different begomoviruses (Table 1) were obtained by searching NCBI GenBank using the protein sequence of ToLCGdV C4. Phylogenetic analysis shows that ToLCGdV C4 shares the highest amino acids similarity (92.94%) with AC4 of *Euphorbia leaf curl virus* (EuLCuV) (Figure 1B), an *Euphorbia pulcherrima*-infecting monopartite begomovirus firstly identified in China (Ma et al., 2004). ToLCGdV C4 is also close to AC4 proteins encoded by other weed-infecting begomoviruses like Asystasia begomovirus 2 (ABgV2), Allamanda leaf curl virus (AllLCV), and Pouzolzia mosaic Guangdong virus (PouMGDV) (Figure 1B). In contrast, ToLCGdV C4 has relatively farther genetic distance with other tomato-infecting begomoviruses (Figure 1B). Thus, it is speculated that ToLCGdV may be a recombinant begomovirus originated from the weed-infecting begomoviruses.

**TABLE 1:** Begomoviruses used in the phylogenetic analyses.

Abbreviation	Name	Accession number		Origin
		DNA A	DNA B	
ABgV2	Asystasia begomovirus 2	JF694486		West Africa
ACMBFV-[BF:Oua:BF127:08]	African cassava mosaic Burkina Faso virus	HE616777		Burkina Faso: Ouagadougou
AllLCV-A[CN:Gd10:06]	Allamanda leaf curl virus	EF602306		China: Guangdong
AYVV	*Ageratum yellow vein virus*	KC810890		China: Hainan
ClGMJsV-[CN-JsXY2-08]	*Clerodendrum golden mosaic Jiangsu virus*	FN396966		China: Jiangsu
EuLCuV-[CN:Fuj:06]	*Euphorbia leaf curl virus*	FJ487911		China: Fujian
LaYVV-[CN:Hn:04]	*Lindernia anagallis yellow vein virus*	AY795900		China: Hainan
MaLCuV-[CN:Gx100:Pap:05]	Malvastrum leaf curl virus	AM260699		China: Guangxi
MiYLCV-[VN:Bin:05]	*Mimosa yellow leaf curl virus*	DQ641695		Vietnam: Binhduong
PaLCuGdV-[CN:Gd2:02]	*Papaya leaf curl Guandong virus*	AJ558122		China: Guangdong
PepYVMLV-[BF:Ban:Hpe2:09]	*Pepper yellow vein Mali virus*	FN555173		Burkina Faso: Banfora
PouMGDV	Pouzolzia mosaic Guangdong virus	NC_038453		China: Guangdong
SaLCV	*Sauropus leaf curl virus*	JN809825		Thailand: Kamphaengsaen
TbLCYnV-CN[CN:Yn136:02]	*Tobacco leaf curl Yunnan virus*	AJ512761		China: Yunnan
ToLCArV-[TZ:Kil:05]	*Tomato leaf curl Arusha virus*	EF194760		Tanzania: Kilimandjaro
ToLCDiV-[MG:Nam:01]	Tomato leaf curl Diana virus	AM701765		Madagascar: Namakely
ToLCGdV	*Tomato leaf curl Guangdong virus*	AY602165		China: Guangdong
ToLCGxV-[CN:Gx1:03]	*Tomato leaf curl Guangxi virus*	AM236784		China: Guangxi
ToLCJaV-A[ID]	*Tomato leaf curl Java virus*	AB100304		Indonesia
ToLCLV-[LA]	*Tomato leaf curl Laos virus*	AF195782		Laos
ToLCMLV-[ML]	*Tomato leaf curl Mali virus*	AY502936		Mali
ToLCMYV-MY[MY:Kla:97]	*Tomato leaf curl Malaysia virus*	AF327436		Malaysia
ToLCPV-A[PH:Lag:06]	*Tomato leaf curl Philippines virus*	AB377113		Philippines: Laguna
ToLCSiV-[NI:SL]	*Tomato leaf curl Sinaloa virus*	AJ608286	AJ508783	Nicaragua: Santa Lucia
ToLCToV-[MG:Mia:01]	Tomato leaf curl Toliara virus	AM701768		Madagascar: Miandrivazo
ToLCV-Sol[AU:Sol:D1]	*Tomato leaf curl virus*	AF084006		Australia
TYLCYnV-[CN:YN2013:11]	*Tomato yellow leaf curl Yunnan virus*	KC686705		China: Yunnan

### C4 Contributes to ToLCGdV Pathogenicity

**FIGURE 2:**
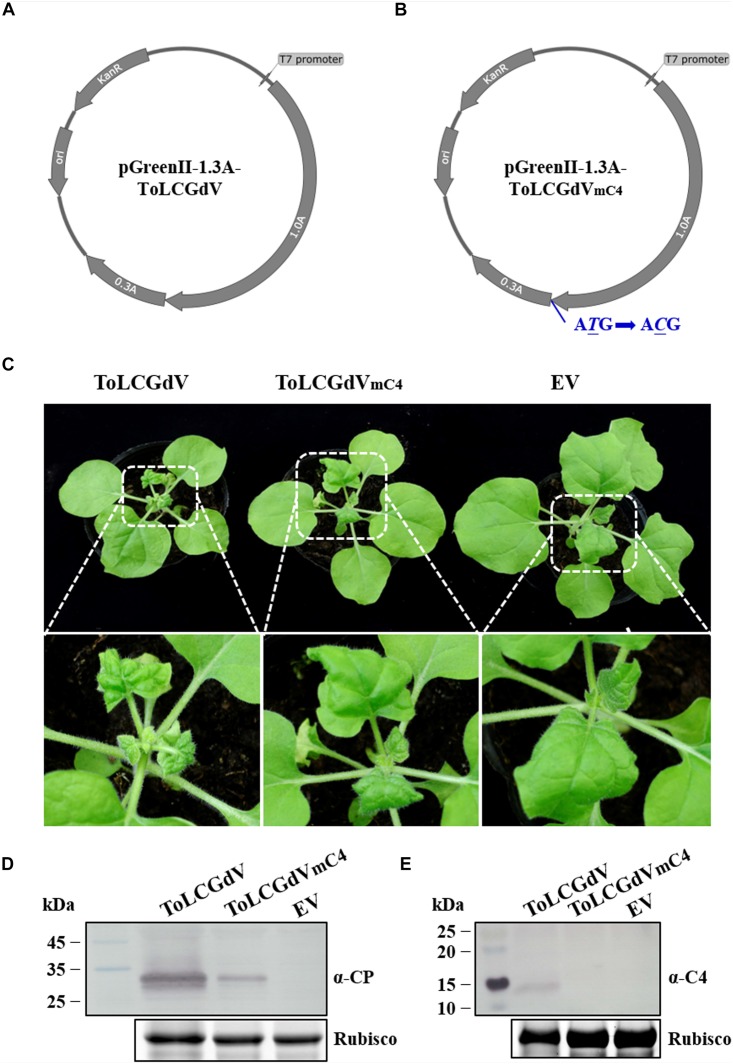
Construction of ToLCGdV and ToLCGdV_mC4_ infectious clones. **(A)** Schematic depiction of pGreenII-1.3A-ToLCGdV. Full-length and 0.3-time sequence of ToLCGdV were amplified and cloned into pGreenII vector (Hellens et al., 2000). *KanR*, kanamycin resistance. *ori*, replication initial origin. **(B)** Schematic depiction of pGreenII-1.3A-ToLCGdV_mC4_. Loss expression of C4 was made by replacing the start codon ATG with ACG, which has no effect on the expression of C1. *KanR*, kanamycin resistance. *ori*, replication initial origin. **(C)** Symptoms of *N. benthamiana* plants agroinfiltrated with pGreenII-1.3A-ToLCGdV or pGreenII-1.3A-ToLCGdV_mC4_. *Agrobacterium* strains harboring pGreenII-1.3A-ToLCGdV or pGreenII-1.3A-ToLCGdV_mC4_ were infiltrated into the leaves of 5–6 leaf-stage *N. benthamiana.* Photos were taken at 13 dpi. The bottom panel shows the magnification of the white dotted frame. EV, empty vector. **(D)** Western blot detection of virus accumulation in the infected plants. The upper leaves were taken at 13 dpi and subjected to western blot detection with anti-ToLCGdV AV1 (CP) antibody. Rubisco shows equal sample loading. Numbers on the left indicate molecular weight. **(E)** Western blot detection of C4 in the upper infected leaves. Leaves were taken at 13 dpi to perform western blot with anti-C4 antibody. Rubisco is used as equal loading. Numbers on the left indicate molecular weight.

To study the molecular characteristic of ToLCGdV, the infectious clones of ToLCGdV and ToLCGdV_mC4_ which contains an unexpressed C4 were constructed. pGreenII-1.3A-ToLCGdV (Figure 2A) and pGreenII-1.3A-ToLCGdV_mC4_ (Figure 2B) were transformed to *Agrobacterium tumefaciens* strain GV3101 and then infiltrated into 5–6 leaf stage *N. benthamiana* plants. Comparing with control plants, plants agroinfiltrated with pGreenII-1.3A-ToLCGdV showed symptoms with stunting, newly leaves curly, and yellowing at 10 days post inoculation (dpi) (Figure 2C), while plants agroinfiltrated with pGreenII-1.3A-ToLCGdV_mC4_ showed visible symptoms with newly leaves curly until 15 dpi (Figure 2C). Western blot detection also confirmed that though the mutant virus can still move systemically to the upper leaves, the virus accumulation reduced significantly due to the loss of C4 (Figures 2D,E). In summary, inoculation of *N. benthamiana* verified the validity of the infectious clones and demonstrate that loss of C4 remarkably delay and weaken disease symptoms of ToLCGdV in *N. benthamiana*.

**FIGURE 3:**
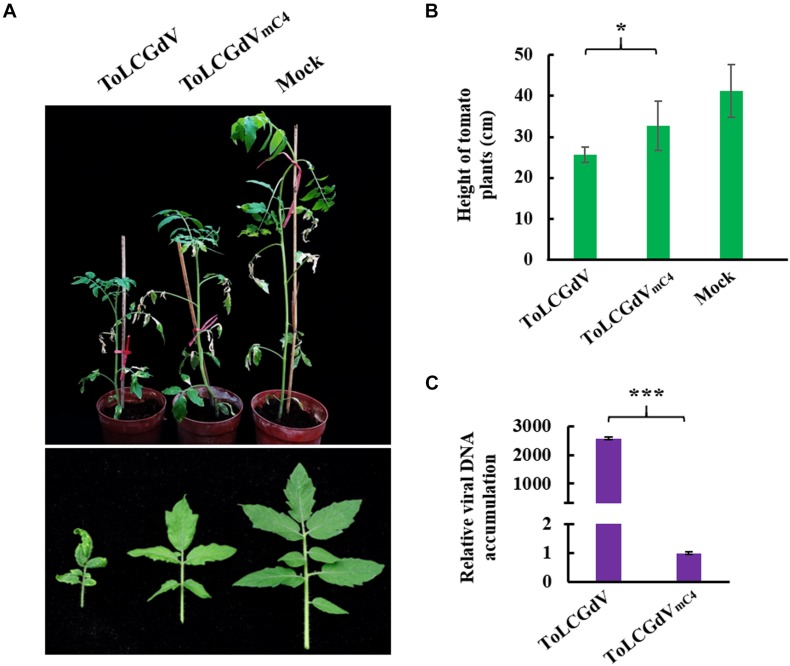
Effects of C4 on ToLCGdV pathogenicity in tomato. **(A)** Symptoms of tomato plants infected by ToLCGdV or ToLCGdV_mC4_. *Agrobacterium* strains harboring pGreenII-1.3A-ToLCGdV or pGreenII-1.3A-ToLCGdV_mC4_ were infiltrated into 4–6-week-old tomato plants. Photos were taken at 30 dpi. **(B)** Statistical analysis of the height of tomato plants infected by ToLCGdV and ToLCGdV_mC4_. Ten plants of each treatment were performed to measure the height. *p < 0.05. **(C)** Quantitative PCR to detect the viral DNA accumulation. Samples were taken at 30 dpi and subjected to DNA extraction. 0.2 μg DNA was used to perform real-time PCR. ***p < 0.001 (extremely significant).

To explore the function of C4 during ToLCGdV infection in its natural host tomato, *Agrobacterium* strain containing pGreenII-1.3A-ToLCGdV or pGreenII-1.3A-ToLCGdV_mC4_ was infiltrated into tomato plants. Tomato plants agroinfiltrated with pGreenII-1.3A-ToLCGdV developed obvious symptoms including leaf curling and yellowing at 30 dpi (Figure 3A), indicating the monopartite nature of ToLCGdV. However, tomato plants infected by ToLCGdV_mC4_ showed much milder symptoms compared with plants infected by ToLCGdV or mock-treated (Figure 3A). In addition, ToLCGdV_mC4_-infected tomato plants were not as shortened as ToLCGdV-infected tomato plants (Figure 3B). Quantitative PCR also confirmed that loss of C4 significantly reduced viral accumulation (Figure 3C). These results implied that though ToLCGdV can still move systemically without C4 expression, C4 plays an important role in ToLCGdV symptom development.

### ToLCGdV C4 Could Enhance PVX Pathogenicity

**FIGURE 4:**
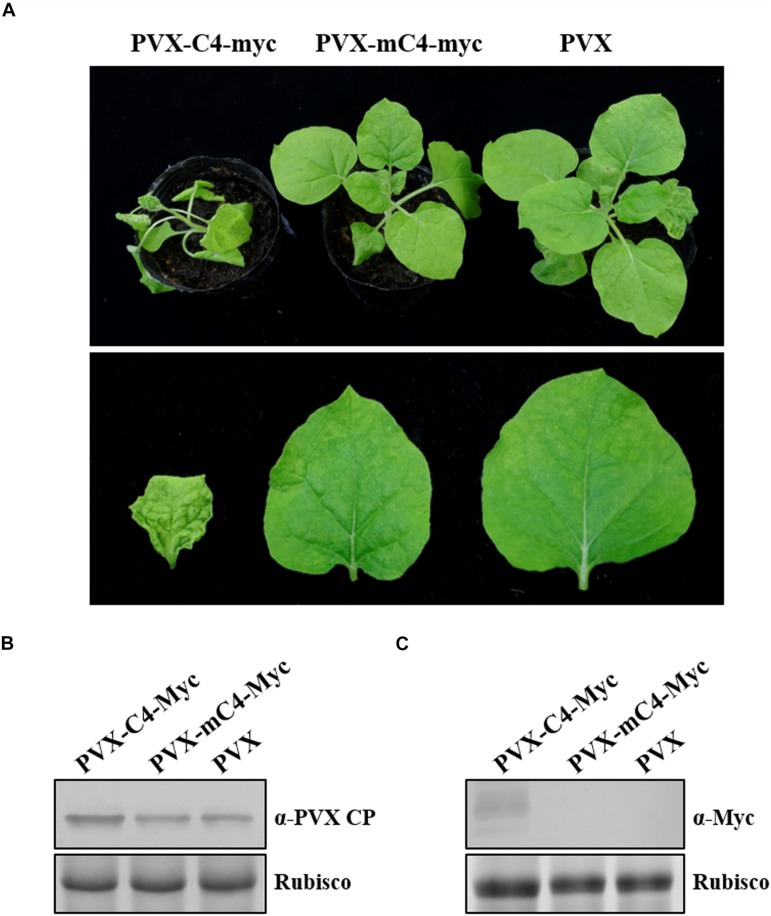
Enhancement of C4 on PVX symptom development. **(A)** Symptoms of *N. benthamiana* plants infected by PVX-C4-Myc, PVX-mC4-Myc, and PVX, respectively. *Agrobacterium* strain harboring PVX-C4-Myc, PVX-mC4-Myc, or PVX was infiltrated into 5–6 leaf-stage *N. benthamiana* plants. Photos were taken at 10 dpi. **(B)** Western blot detection of the upper infected leaves. Samples were taken at 10 dpi and subjected to western blot with anti-PVX CP antibody. Rubisco indicates equal sample loading. **(C)** Western blot to detect C4 expression. Samples were taken at 10 dpi and western blot was performed with anti-Myc antibody. Rubisco indicates equal sample loading.

To further substantiate the role of C4 in pathogenesis independent of ToLCGdV, C4 fused with Myc tag was cloned into the *Potato virus X* (PVX) vector pGR107 (Lu et al., 2003) to yield PVX-C4-Myc. Moreover, mC4-Myc with start codon mutation was also cloned into pGR107 to make PVX-mC4-Myc. *Agrobacterium* containing PVX-C4-Myc, PVX-mC4-Myc, or PVX was infiltrated into 5–6 leaf-stage *N. benthamiana*. Mild mosaic symptoms on the top leaves of *N. benthamiana* infected by PVX-C4-Myc were first observed at 5 dpi, while no obvious symptoms were observed on plants inoculated with PVX-mC4-Myc or PVX at this time (Supplementary Figure 1). At 10 dpi, severe mosaic and curling of the leaves were observed on plants infected by PVX-C4-Myc, while only mild mosaic was observed in plants infected by PVX-mC4-Myc or PVX (Figure 4A). At later time, symptoms on the newly emerged leaves of plants inoculated with PVX or PVX-mC4-Myc became very weak and hardly visible (Supplementary Figure 1). However, symptoms including mosaic, curling, and distortion caused by PVX-C4-Myc sustained throughout the life of the plants. Western blot experiments performed at 10 dpi with anti-PVX CP antibody and anti-Myc antibody confirmed that C4 could remarkably increase PVX accumulation (Figures 4B,C), further implying that C4 may be a pathogenic determinant independent of ToLCGdV infection.

### ToLCGdV C4 Suppresses PTGS by Repressing Systemic Gene Silencing but Not Local Gene Silencing

**FIGURE 5:**
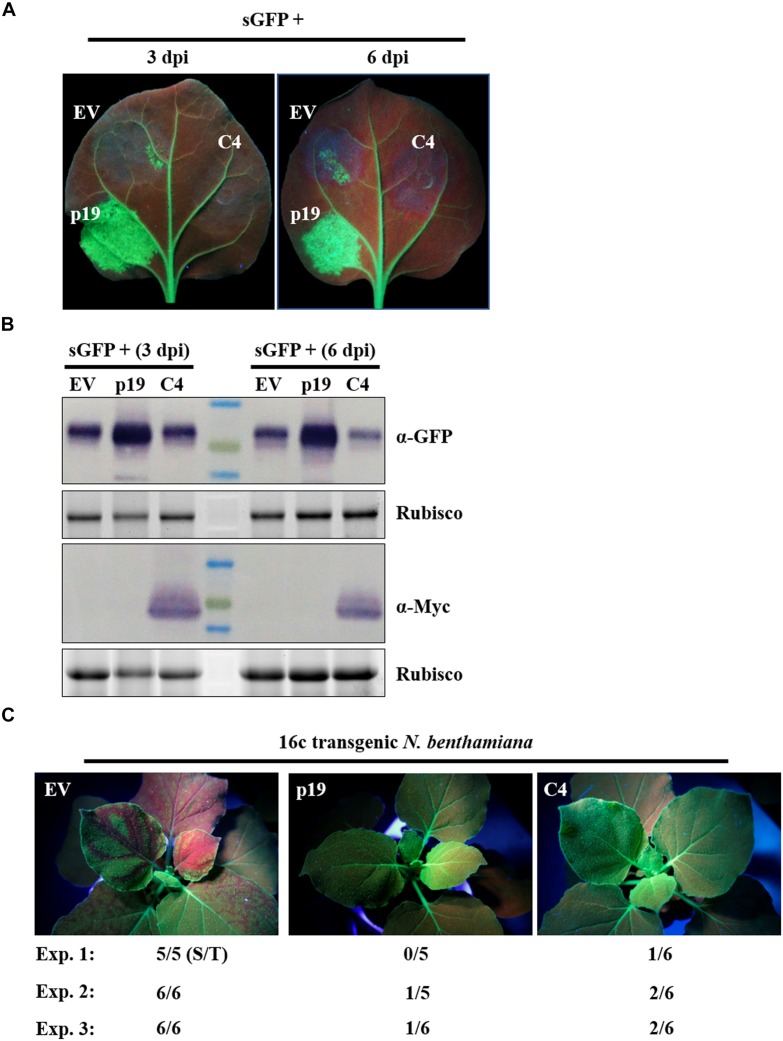
Suppression of ToLCGdV C4 in PTGS. **(A)** Effect of C4 in local gene silencing. *Agrobacterium* strains containing sGFP and p19, sGFP and EV, or sGFP and C4-Myc were co-infiltrated into the same leaf of *N. benthamiana*. EV and p19 were used as negative and positive controls, respectively. Leaves were photographed under UV light at 3 and 6 dpi. **(B)** Western blot assays of GFP and C4 expression in co-infiltrated leaves. Samples taken at 3 and 6 dpi were subjected to western blot with anti-GFP and anti-Myc antibody. Rubisco indicates equal sample loading. **(C)** Suppression of C4 in systemic gene silencing. *Agrobacterium* strains containing sGFP and p19, sGFP and EV, or sGFP and C4-Myc were co-infiltrated into 16c transgenic *N. benthamiana* plants. Inoculated plants were observed under UV light at 12 dpi. Three independent experiments were repeated. The number ratio (S/T) indicates the systemic silencing (S) among the total number of infiltrated plants (T).

AC4/C4 encoded by some begomoviruses have been reported to function as a VSR (Vanitharani et al., 2004; Bisaro, 2006; Gopal et al., 2007). To explore the function of ToLCGdV C4 in RNA silencing suppression, a widely adopted method based on *Agrobacterium* infiltration was utilized (Johansen, 2001). *Agrobacterium* strains harboring C4-Myc and the positive sense-GFP (sGFP) (Bragg and Jackson, 2004; Dong et al., 2016; Zhang et al., 2017, 2018) were mixed equally and co-infiltrated into *N. benthamiana* leaves. Co-infiltration of p19 and sGFP was used as positive control, while co-infiltration of empty vector (EV) and sGFP was used as negative control. Three days after agroinfiltration, strong GFP fluorescence was observed in regions co-infiltrated with sGFP and p19 (Figure 5A). However, no obvious or little fluorescence was observed in leaf patches co-infiltrated with sGFP and C4-Myc or sGFP and EV (Figure 5A). The results were the same at 6 dpi. Western blot detection confirmed that C4 had no effect on GFP protein accumulation compared with p19 and EV (Figure 5B).

Next, we tested whether C4 suppresses systemic movement of RNA silencing signals. C4-Myc, p19, or EV were mixed with sGFP and co-infiltrated into leaves of 16c transgenic *N. benthamiana* (Voinnet and Baulcombe, 1997). Three independent experiments were repeated to detect the systemic movement of GFP silencing signals. As expected, transient expression of p19, but not the EV, suppressed the movement of GFP silencing signals from infiltrated leaves to upper leaves at 12 dpi (Figure 5C). C4 could also suppressed systemic movement of GFP silencing signals, though not as effective as p19 (Figure 5C).

In summary, these results demonstrate that C4 functions as a VSR by repressing systemic gene silencing but not local gene silencing.

### ToLCGdV C4 Suppresses Methylation-Mediated TGS

**FIGURE 6:**
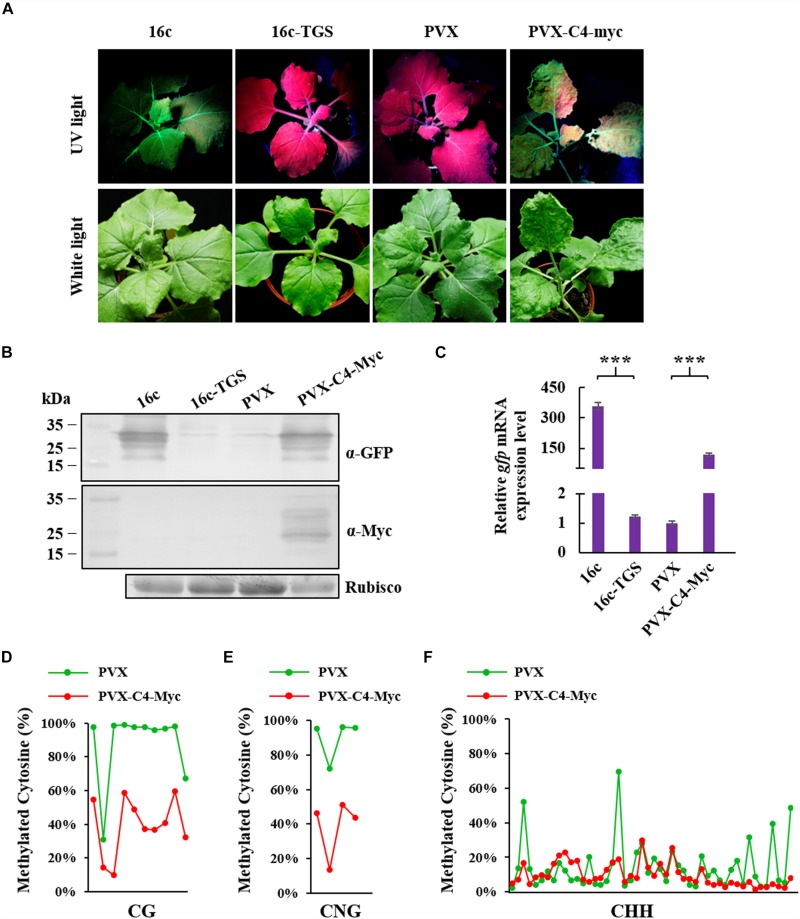
Suppression of ToLCGdV C4 in TGS. **(A)** PVX-based expression of C4 suppressed TGS. *Agrobacterium* strains containing sGFP and p19, sGFP and EV, or sGFP and C4-Myc were co-infiltrated into 16c-TGS *N. benthamiana* plants. At 12 dpi, the infiltrated plants were observed and photographed under UV light and white light. **(B)** Western blot assays to detect the expressions of GFP and C4 in infiltrated plants. Samples were taken at 12 dpi and subjected to western blot with anti-GFP and anti-Myc antibody, respectively. Rubisco was used as a loading control. Numbers on the left indicate molecular weight. **(C)** Real-time RT-PCR detection of GFP mRNA accumulation in infiltrated 16c-TGS plants. Samples were taken at 12 dpi and 2 μg RNA was used to perform detection. ^∗∗∗^*p* < 0.001 (extremely significant). Cytosine methylation level of CG sites **(D)**, CNG sites **(E)**, and CHH sites **(F)** in 35S promoter region. Cytosine methylation level was measured by next generation sequencing-based bisulfite sequencing PCR (BSP). Green dots represent the cytosine residues of 35S promoter in 16c-TGS plants infiltrated with PVX, and red dots represents the cytosine residues of 35S promoter in plants infiltrated with PVX-C4-Myc. The detailed methylation level of each CG, CNG, and CHH sites are listed in Supplementary Table 2.

To identify whether ToLCGdV C4 could suppress methylation-mediated TGS, 16c-TGS transgenic *N. benthamiana* plants were used. In 16c-TGS plants, GFP was silenced by TGS, and TGS was induced by *Tobacco rattle virus* (TRV) vector which contains a portion of the 35S promoter sequence (Buchmann et al., 2009; Raja et al., 2010). *Agrobacterium* harboring PVX-C4-Myc or PVX was infiltrated into 16c-TGS plants, and the plants were photographed under white light and UV lamp at 12 dpi. 16c-TGS plants infiltrated with PVX showed no visible GFP fluorescence, while plants infiltrated with PVX-C4-Myc showed obvious GFP fluorescence (Figure 6A). 16c and 16c-TGS *N. benthamiana* plants were used as positive and negative controls, respectively. Western blot and real-time RT-PCR confirmed that GFP protein and mRNA expressions were significantly increased in 16c-TGS plants infiltrated with PVX-C4-Myc compared with plants infiltrated with PVX (Figures 6B,C).

To further confirm the ability of C4 to reverse methylation-mediated TGS, next generation sequencing-based bisulfite sequencing PCR (BSP) was performed to assess the cytosine methylation level of 35S promoter. Cytosine methylation level of ten CG, four CNG, and forty-eight CHH sites in the 35S promoter region were analyzed. C4 expressed from the PVX vector reduced cytosine methylation remarkably at CG (48%), CNG (51%), and CHH (5%) sites (Figures 6D–F and Supplementary Table 2). These results indicated that ToLCGdV C4 is able to suppress methylation-mediated TGS.

### C4 Colocalizes and Interacts With BAM1

**FIGURE 7:**
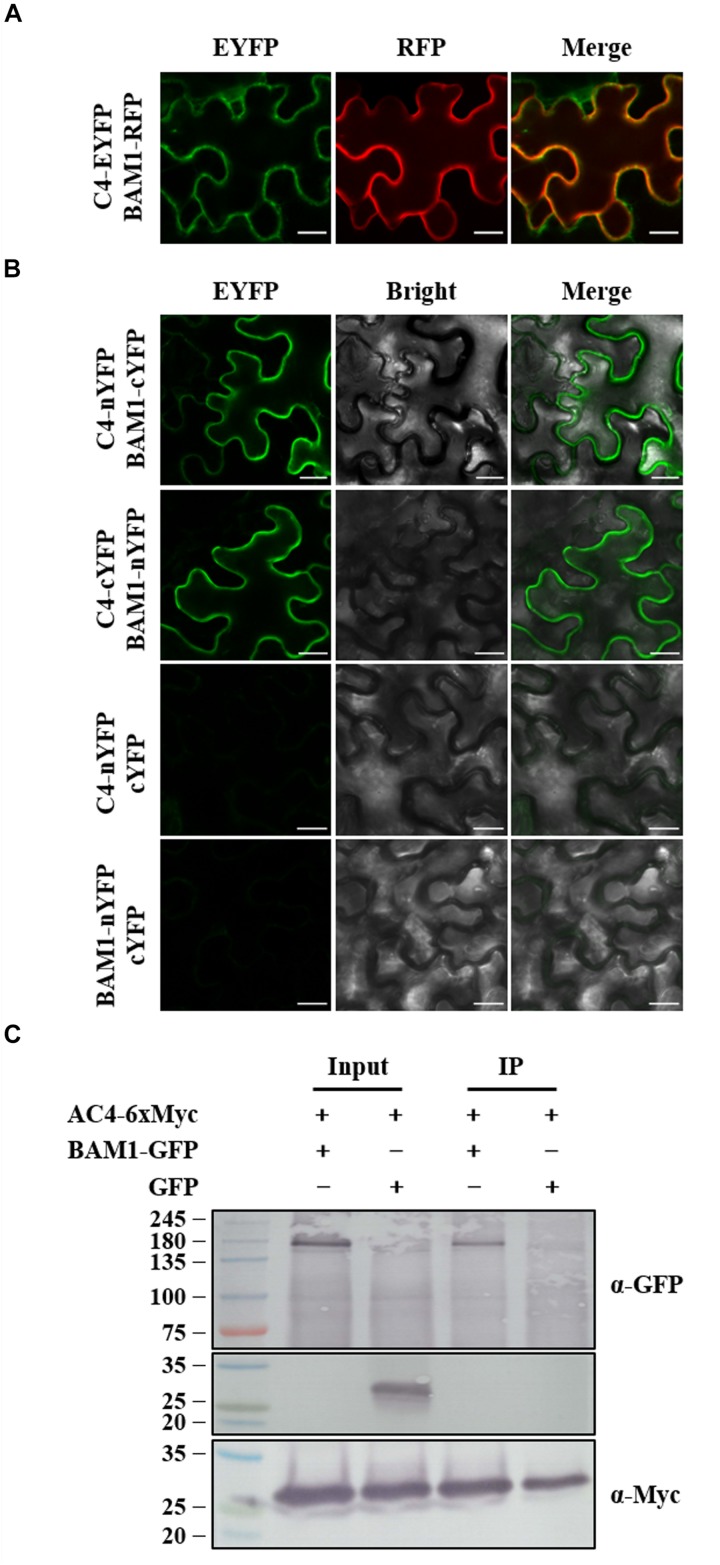
Co-localization and interaction between C4 and BAM1. **(A)** Co-localization of C4 and BAM1. *Agrobacterium* strains harboring C4-EYFP and BAM1-RFP were co-infiltrated into *N. benthamiana* leaves. At 3 dpi, the infiltrated leaves were taken and observed under confocal microscope. Bars = 20 μm. **(B)** Interaction between C4 and BAM1 by BiFC. *Agrobacterium* strains harboring C4-nYFP and BAM1-cYFP, or C4-cYFP and BAM1-nYFP were co-infiltrated into *N. benthamiana* leaves and subjected to confocal microscopy observation at 3 dpi. C4-nYFP and cYFP, and BAM1-nYFP and cYFP were used as negative controls. Bars = 20 μm. **(C)** Co-IP assays to detect the interaction between C4 and BAM1. *Agrobacterium* strains containing AC4-6xMyc and BAM1-GFP, or AC4-6xMyc and GFP were co-infiltrated into *N. benthamiana* leaves. Samples were taken at 3 dpi to perform Co-IP assays. Western blot detections were conducted with anti-GFP and anti-Myc antibodies, respectively. Numbers on the left indicate molecular weight.

*Tomato yellow leaf curl virus* C4 and MYMV AC4 have been reported to interact with BAM1 in PM and plasmodesmata (PD) to inhibit the intercellular spread of RNAi (Carluccio et al., 2018; Rosas-Diaz et al., 2018), the interaction between ToLCGdV C4 and BAM1 was also investigated. *Agrobacterium* strains containing C4-EYFP and BAM1-RFP were co-infiltrated into *N. benthamiana* leaves. C4 co-localizes with BAM1 both in PM and PD (Figure 7A). BiFC assays found that C4 interacts with BAM1 at PM (Figure 7B). Co-IP assays further confirmed the interaction between C4 and BAM1 (Figure 7C). These results imply that ToLCGdV C4 may adopt the same strategy as TYLCV C4 to suppress PTGS (Rosas-Diaz et al., 2018).

## Discussion

In this study, we report that ToLCGdV may be a recombinant begomovirus originated from some weed-infecting begomoviruses based on phylogenetic analyses of multiple AC4/C4 protein sequences. ToLCGdV C4 is closely related to the AC4 of *Euphorbia leaf curl virus* (EuLCuV), Asystasia begomovirus 2 (ABgV2), Allamanda leaf curl virus (AllLCV), and Pouzolzia mosaic Guangdong virus (PouMGDV). All these begomoviruses are firstly isolated from wild plant hosts (He et al., 2009; Tang et al., 2014; Wyant et al., 2015), demonstrating the frequent recombination between wild hosts and crops (Lefeuvre and Moriones, 2015; Garcia-Arenal and Zerbini, 2019). Recombination is a key process in the evolution of many viruses, especially for geminiviruses which contain a highly compacted genome (∼2.7 kb) (Garcia-Andres et al., 2007; Varsani et al., 2008; Martin et al., 2011). It has been highly speculated that recombination between different geminiviruses in even different hosts is one of the main reasons for the diversification of geminiviruses (Padidam et al., 1999; Briddon et al., 2010). One of the best studied examples is the recombination between TYLCD-associated begomoviruses. *Begomovirus* contains over 320 species and is one of the largest plant virus genera (Zerbini et al., 2017). Begomoviruses are transmitted by whitefly between wild hosts and crops (Perefarres et al., 2012; Rey et al., 2012), which increases the opportunities of mix infection and recombination (Lima et al., 2012). *Tomato leaf curl Yunnan virus* (TLCYnV), a begomovirus firstly isolated from *Malvastrum coromandelianum*, evolved from *Tomato yellow leaf curl China virus* (TYLCCNV) by recombination and is highly infectious to a range of host plants by acquiring a more virulent C4 (Xie et al., 2013). Moreover, a recombinant virus named tomato yellow leaf curl Malaga virus (TYLCMalV) is the recombination of *Tomato yellow leaf curl Sardinia virus* (TYLCSV) and TYLCV (Monci et al., 2002). TYLCMalV exhibits a novel pathogenic phenotype and has an enlarge host range, which contribute to the prevalence in the region where it was detected (Monci et al., 2002). Thus, we speculate that ToLCGdV may be a recombinant virus evolved from two or more wild plant hosts, acquiring more virulence and becoming prevalent in Guangdong province where ToLCGdV was isolated.

We also found that disruption of C4 delayed ToLCGdV symptom development in *N. benthamiana* and significantly reduced viral DNA accumulation in tomato plants. However, the detailed function of C4 during ToLCGdV infection remains unknown. C4 has long been demonstrated to be required for monopartite begomovirus infection (Stanley and Latham, 1992; Teng et al., 2010; Li et al., 2018). Recently, AC4 of EACMCV has been shown to be involved in virus infection, knockout mutation in AC4 ORF delayed virus symptom development and plants recovered from the mutant virus infection (Chen et al., 2019). In addition, AC4 of ACMV (Hipp et al., 2016) and of MYMV (Carluccio et al., 2018) were also required for virus infection.

*Tomato leaf curl Guangdong virus* C4 expressed from the PVX vector significantly increased PVX concentration, and induced leaf curling and severe mosaic in plants inoculated with PVX-C4-Myc compared with plants inoculated with PVX or PVX-mC4-Myc. This result implies that C4 may be a pathogenic determinant essential for abnormalities in infected plants. Induction of hyperplasia and tumorigenic growth in infected plant tissues by AC4/C4 has been extensively studied (Mills-Lujan et al., 2015). The nature of hyperplasia and tumorigenic growth is uncontrolled DNA replication and loss function of host cell cycle regulators. AC4/C4 is considered to be the “oncogene” that manipulates the host cell cycle to stimulate DNA replication (Nikitin and Luftig, 2012). However, the mechanism about how AC4/C4 induces abnormalities in infected plants may be variable according to the diversity of AC4/C4. A recent study found that TLCYnV C4 interacts and relocates the glycogen synthase kinase 3 (GSK3)/SHAGGY like kinase, named NbSKη, from the nucleus to membrane. Relocalization of NbSKη affects the degradation of Cyclin D1.1, thereby inducing the cell division (Mei et al., 2018b). Another study found that *Beet severe curly top virus* (BSCTV) C4 induces the expression of a RING finger E3 ligase, RKP, which antagonizes with an inhibitor of cyclin-dependent kinase (CDK) to induce cell cycle (Cheng et al., 2013).

*Tomato leaf curl Guangdong virus* C4 is a VSR which inhibits both TGS and PTGS. TGS and PTGS are the main pathways exploited by plants to counter virus infection by inducing viral genome methylation or sequence-specific mRNA degradation. TGS acts as defenses against DNA viruses, however, geminiviruses have evolved to encode proteins to interfere with these processes. VSR encoded by begomovirus is often able to suppress DNA methylation-mediated TGS, like βC1 of Tomato yellow leaf curl China virus betasatellite (TYLCCNB) (Yang et al., 2011), C4 of CLCuMuV (Ismayil et al., 2018), and C4 of *Tomato leaf curl Yunnan virus* (Xie et al., 2013). TYLCCNB βC1 interacts and inhibits the activity of a key enzyme required for maintenance of the methyl cycle, S-adenosyl homocysteine hydrolase (SAHH) (Yang et al., 2011).

*Tomato leaf curl Guangdong virus* C4 inhibits systemic gene silencing but not local gene silencing, this is also the case for TYLCV C4 (Luna et al., 2012). AC4/C4 of TYLCV or MYMV has been found to suppress intercellular spread of RNAi by interacting with BAM1 (Rosas-Diaz et al., 2018). We found that ToLCGdV C4 also colocalizes and interacts with BAM1. However, AC4/C4 encoded by TYLCV, MYMV, and ToLCGdV shares low amino acids identity (Supplementary Figure 2), implying that different begomoviruses may adopt the same strategy to suppress PTGS.

## Data Availability Statement

All datasets generated for this study are included in the article/Supplementary Material.

## Author Contributions

ZL, ZD, and ZH conceived and designed the experiments. ZL, ZD, YT, and LY conducted the experiments. XS, XW, GL, and YZ analyzed the data. ZL and ZD wrote the manuscript. All authors read and approved the manuscript.

## Conflict of Interest

The authors declare that the research was conducted in the absence of any commercial or financial relationships that could be construed as a potential conflict of interest.

## Supplementary Material

FIGURE S1 Symptoms of *N. benthamiana* infected by PVX-C4-myc, PVX-mC4-myc, and PVX at different times.

FIGURE S2 Amino acids alignment of AC4/C4 of ToLCGdV, TYLCV, and MYMV.

TABLE S1 Primers used in this study.

TABLE S2 Cytosine methylation level of each CG, CNG, and CHH site.
