# Supplementary material for: C4, the Pathogenic Determinant of Tomato Leaf Curl Guangdong Virus, May Suppress Post-transcriptional Gene Silencing by Interacting With BAM1 Protein
Source: Front Microbiol. 2020 May 5;11:851. doi: 10.3389/fmicb.2020.00851 (PMC7215500; doi:10.3389/fmicb.2020.00851)
Supplement: FIGURE S1 — Symptoms of N. benthamiana infected by PVX-C4-myc, PVX-mC4-myc, and PVX at different times. [file Presentation_1.pdf]

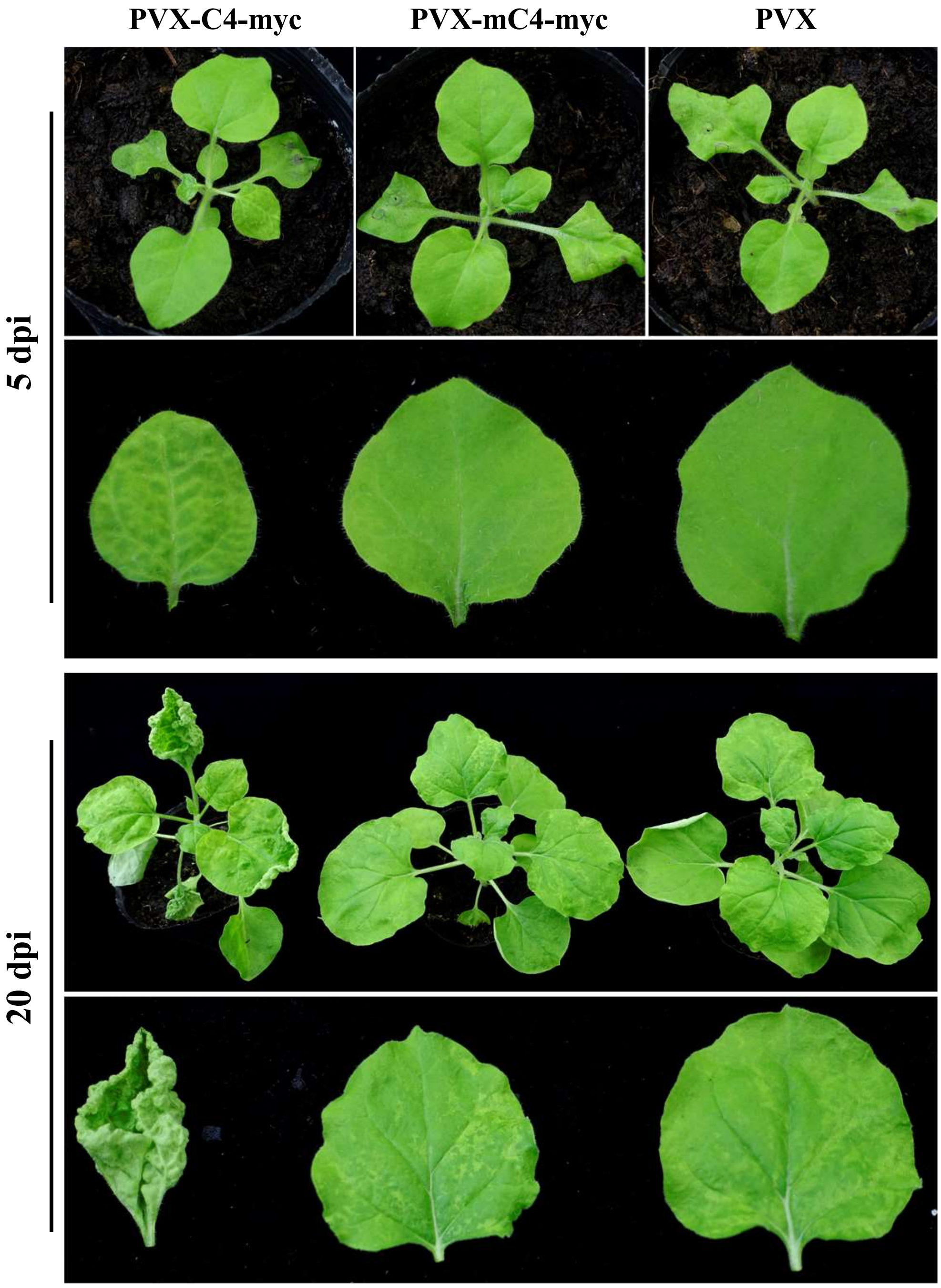

Supplementary Figure 1 Symptoms of *N. benthamiana* infected by PVX-C4-myc, PVX-mC4-myc, and PVX at different times.

```
ToLCGdV C4   ..MGTLISTCLCNSKANTTARITDSSTWFP   28
TYLCV C4     ..MGNHISMCLSNSKANTNVRTNGSSTWYP   28
MYMV AC4     MKMENLISMFCFNSKGSSKRRTQGSSTWFP   30

ToLCGdV C4   QPDQHISIRTFRELNPAPMSSPTSIRMETS   58
TYLCV C4     QTGQHISIRTFRQLRAQQMSRPTWRKTETS   58
MYMV AC4     QQDQHITIRTFRQLKAHQMLSHTWTKTETS   60

ToLCGdV C4   LNGVNSRSTDEVLGEAARMLTTHVQRL...   85
TYLCV C4     LILEFPKSMADQLEEVSNLPTTHMPKHSIQ   88
MYMV AC4     LTMEVSKSMADQLEEVNSLPTTLMPRHSIV   90

ToLCGdV C4   ..........                       85
TYLCV C4     AVNPRPSIY                        97
MYMV AC4     DPSYRPSIY                        99
```

Supplementary Figure 2 Amino acids alignment of AC4/C4 of ToLCGdV, TYLCV, and MYMV

The GenBank accession numbers are AAU00332 (ToLCGdV C4) CAA33691 (TYLCV C4), and QDN53931 (MYMV AC4)
