# Supplementary material for: C4, the Pathogenic Determinant of Tomato Leaf Curl Guangdong Virus, May Suppress Post-transcriptional Gene Silencing by Interacting With BAM1 Protein
Source: Front Microbiol. 2020 May 5;11:851. doi: 10.3389/fmicb.2020.00851 (PMC7215500; doi:10.3389/fmicb.2020.00851)
Supplement: TABLE S1 — Primers used in this study. [file Data_Sheet_1.PDF]

Supplementary Material

C4, the pathogenic determinant of *Tomato leaf curl Guangdong virus*, may suppress post-transcriptional gene silencing by interacting with BAM1 protein

Zhenggang Li[1, 2], Zhenguo Du[1], Yafei Tang[1], Xiaoman She[1], Xiaomei Wang[1], Yanhua Zhu[1], Lin Yu, Guobing Lan, Zifu He[1, 2]*

*** Correspondence:** Zifu He: hezf@gdppri.com

[1] Plant Protection Research Institute, Guangdong Academy of Agricultural Sciences, Guangzhou, 510640, P. R. China

[2] Guangdong Provincial Key Laboratory of High Technology for Plant Protection, Guangzhou, 510640, P. R. China

Supplementary Table 1 | Primers used in this study

**Primer**	**Primer sequence (5'-3')** [a, b]	**Purpose**
ToLCGdV-F1	ATATCGAATTCCTGCAGCCCTCTTGTGTAATTCTCTGCAAATTTTTATGA	pGreenII-1.3A-ToLCGdV
ToLCGdV-R1	TTACACAAGAATGGGAACCCTCATCTCC	
ToLCGdV-F2	GGGTTCCCATTCTTGTGTAATTCTCTGCAAATTTTTATG	
ToLCGdV-R2	CTAGAACTAGTGGATCCCCCTTCACAACCCCGAGGAAC	
C4-ACG-F	GGAGATGAGGGTTCCC***G***TTCTTGTGTAA	pGreenII-1.3A-ToLCGdV$_{mC4}$
C4-ACG-R	TTACACAAGAA***C***GGGAACCCTCATCTCC	
PVX-C4-F	CAGCTAGC**ATCGAT**TCCCGGGATGGGAACCCTCATCTCCACG	PVX-C4
PVX-mC4-F	CAGCTAGC**ATCGAT**TCCCGGGACGGGAACCCTCATCTCCACG	PVX-mC4
PVX-C4-R	CTTATCGGCG**GTCGAC**CCGGGTTAAAGCCTCTGCACATGCGT	
C4-Myc-F	CCG**CTCGAG**ATGGGAACCCTCATCTCCACG	pGD-C4-Myc
C4-Myc-R	GG**GGGCCC**GCAAGCCTCTGCACATGCGTCG	

BiFC-C4-F	GG**ACTAGT**ATGGGAACCCTCATCTCCA	35SNE-C4<br>35SCE-C4
BiFC-C4-R	CG**GGATCC**AAGCCTCTGCACATGCGTCG	
pGDGm-C4-F	CCG**CTCGAG**ATGGGAACCCTCATCTCCA	pGDGm-C4
pGDGm-C4-R	GG**GGGCCC**AAGCCTCTGCACATGCGTCG	

[a] Underlined and bold letters indicate the sequences of the restriction sites.

[b] Underlined, italic, and bold letters indicate the sequences altered by site-specific mutagenesis

Supplementary Table 2 | Cytosine methylation level of each CG, CNG, and CHH site

	PVX			PVX-C4-Myc		
**Relative position of 35S promoter**	**Methylated cytosine reads**	**Non-methylated cytosine reads**	**Ratio of methylated cytosine**	**Methylated cytosine reads**	**Non-methylated cytosine reads**	**Ratio of methylated cytosine**
**CG**						
121	63841	1694	**0.974**	35639	29896	**0.544**
139	20304	45231	**0.31**	9529	56006	**0.145**
157	64582	953	**0.985**	6337	59198	**0.097**
189	64793	742	**0.989**	38349	27186	**0.585**
199	63800	1735	**0.974**	31888	33647	**0.487**
214	64070	1465	**0.978**	24357	41178	**0.372**
225	62769	2766	**0.958**	23983	41552	**0.366**
265	63375	2160	**0.967**	26660	38875	**0.407**
277	64146	1389	**0.979**	39123	26412	**0.597**
297	44084	21451	**0.673**	21070	44465	**0.322**
**CNG**						
153	62451	3084	**0.953**	30483	35052	**0.465**

156	47281	18254	**0.721**	8697	56838	**0.133**
160	62982	2553	**0.961**	33519	32016	**0.511**
261	62850	2685	**0.959**	28644	36891	**0.437**
**CHH**						
100	1576	63959	**0.024**	3112	62423	**0.047**
102	8824	56711	**0.135**	4507	61028	**0.069**
103	34102	31433	**0.52**	10884	54651	**0.166**
106	8585	56950	**0.131**	2795	62740	**0.043**
112	2727	62808	**0.042**	5491	60044	**0.084**
113	4294	61241	**0.066**	6360	59175	**0.097**
116	7795	57740	**0.119**	5539	59996	**0.085**
135	4478	61057	**0.068**	10671	54864	**0.163**
136	11022	54513	**0.168**	13690	51845	**0.209**
150	8173	57362	**0.125**	14874	50661	**0.227**
151	4422	61113	**0.067**	11335	54200	**0.173**
167	4989	60546	**0.076**	11762	53773	**0.179**
168	3287	62248	**0.05**	4668	60867	**0.071**
169	13180	52355	**0.201**	3675	61860	**0.056**
179	2923	62612	**0.045**	4857	60678	**0.074**
180	2635	62900	**0.04**	5114	60421	**0.078**
181	4223	61312	**0.064**	8277	57258	**0.126**

182	10860	54675	**0.166**	11231	54304	**0.171**
183	45688	19847	**0.697**	12478	53057	**0.19**
185	2354	63181	**0.036**	3828	61707	**0.058**
186	4270	61265	**0.065**	5934	59601	**0.091**
187	14859	50676	**0.227**	5287	60248	**0.081**
196	18434	47101	**0.281**	19433	46102	**0.297**
218	7322	58213	**0.112**	9253	56282	**0.141**
219	12702	52833	**0.194**	6135	59400	**0.094**
222	8579	56956	**0.131**	10755	54780	**0.164**
223	4139	61396	**0.063**	6750	58785	**0.103**
228	15223	50312	**0.232**	16591	48944	**0.253**
231	10098	55437	**0.154**	7612	57923	**0.116**
236	7980	57555	**0.122**	4892	60643	**0.075**
256	2673	62862	**0.041**	5029	60506	**0.077**
258	2061	63474	**0.031**	3643	61892	**0.056**
259	13451	52084	**0.205**	8717	56818	**0.133**
279	6075	59460	**0.093**	3476	62059	**0.053**
281	8208	57327	**0.125**	2475	63060	**0.038**
285	4242	61293	**0.065**	3205	62330	**0.049**
286	2482	63053	**0.038**	1925	63610	**0.029**
287	8394	57141	**0.128**	3627	61908	**0.055**

289	11923	53612	**0.182**	3029	62506	**0.046**
293	2927	62608	**0.045**	2000	63535	**0.031**
294	20548	44987	**0.314**	3680	61855	**0.056**
299	5740	59795	**0.088**	927	64608	**0.014**
304	2180	63355	**0.033**	1610	63925	**0.025**
305	2008	63527	**0.031**	1821	63714	**0.028**
306	25800	39735	**0.394**	2928	62607	**0.045**
309	4374	61161	**0.067**	2188	63347	**0.033**
310	3529	62006	**0.054**	1519	64016	**0.023**
312	31899	33636	**0.487**	5192	60343	**0.079**
